# Re-orienting the Model of Care: Towards Accountable Care Organizations

**DOI:** 10.5334/ijic.4162

**Published:** 2018-04-11

**Authors:** Alexander Pimperl

**Affiliations:** 1 OptiMedis AG, DE

**Keywords:** accountable care organizations, value based purchasing, Outcome based commissioning, health system disruption, integrated care systems, integrated care policy

Ideally, high performing health systems will try to achieve the ‘Triple Aim’ of improving population health, enhancing the individual care experience and doing so in a cost efficient way [1]. In reality, developed countries all over the world face challenges to focus their health care actors on these aims. A major reason for that is the financial and organizational fragmentation of health service delivery. This historic institutional and financial fragmentation of health service delivery in the U.S., Germany, and other European countries, has triggered a variety of issues in these health systems. These problems include gaps in information exchange, lack of coordination between sectors, poor financial incentives for unquantifiable service expansions, missing common goals and values, and poor assignment of functions and positions within the care process. These current and rising problems are associated with unnecessary quality risks for patients, economic inefficiency and disparities in performance and health outcomes [2].

A possible solution is seen in Accountable Care Organizations (ACOs) that seek to integrate providers and services to generate value for defined populations. Established in the US in 2010 through the Centers for Medicare and Medicaid Services (CMS) [3], ACOs have seen rapid proliferation. In just three years, 447 ACOs (CMS & commercial) were implemented covering about 14 million beneficiaries (Q1 2013). By the same period in 2017 some 923 ACOs covering an estimated 32.4 million lives within 1366 ACO contracts had emerged. This accounts for a penetration of approximately 10% of all beneficiaries. Even though CMS triggered this development, commercial contracts tend to be larger. Taken together they encompass 19.4 million people, whereas CMS ACO contracts include 13.3 million lives [4]. Whereas the US has seen a proliferation of ACOs, ACO implementation in Europe is still in its infancy with only selected flagship projects, such as Ribera Salud in Spain or Gesundes Kinzigtal in Germany [5], having any level of maturity.

In the US, ACO reforms on a whole have led to improvements in care quality, while generating cost savings at the same time, thus benefiting payers and the beneficiaries served by these ACOs in the US. Collectively, CMS ACOs produced $836 million in savings, resulting in net savings of $71.4 million for CMS in 2016. Performance data of each CMS ACO contract are made available publicly by CMS. Performance results of commercial contracts are not published in general, but for specific cases evaluation exist. One of the best researched models, is the Alternative Quality Contract in Massachusetts, a precursor and inspiration for the CMS ACO models. Song et al [6] found gross savings of about 7% on average over 4 years and 13,2% in the 4th intervention year. In Europe, for the above mentioned ACO models, Ribera Salud and Gesundes Kinzigtal also realized cost savings of between 6–25% in combination with quality improvements and better health outcomes [5,7].

However, the outcomes of individual ACOs on quality and costs varies widely. Preliminary results from the US suggest that more experienced, physician-led, integrated and smaller ACOs have a greater likelihood of achieving better results. Higher financial benchmarks were associated with greater savings [4]. ACOs serving high proportions of racial and ethnic minorities struggle with quality performance [8].

## Core Principles of Successful ACO Models

Preliminary results examining ACO models suggest some common core principles associated with successful adoption:

*Policy context*: The differences between the growth of ACO models in the US vs. Europe triggers a question. It may be asked, what the reason for the disruptive shift of reorienting the model of care towards ACOs in the US health care practice is, whereas in Europe the call for accountable care seems to be huge, but nothing is really happening in practice. From the perspective of the payers the cost and quality results speak for the way where the ACO reforms are heading. And from the providers point of view? The era of fee-for-service-remuneration seems to come to an abrupt end as a whole in the US. CMS has set a new tone for the whole market with the ACO models and additional alternative payment models, such as bundled-payments and other approaches to link fee-for-service payments to quality. The “target is to have 30% of Medicare payments tied to quality or value through alternative payment models [such as ACOs] by the end of 2016, and 50% of payments by the end of 2018.” [9]. And also the rest of fee-for-service payments (90%) should be tied to value until 2018. For providers this means that they have to act pro-actively or they will very probably loose ground in the healthcare market in this change to value based payment. In European countries such a clear and strong commitment to change to a value based payment system is missing. In addition, there is, so far, a lack of payment models with upfront investment or advance payments for providers to build a financial foundation for the incremental change necessary for value-based approaches in Europe. The lack of ACOs in Europe may be attributed to these policy contexts, as well as an insufficient legal framework to allow payers to set up ACO contracts with the contract characteristics, described below.

*Contract characteristics*: ACOs need to invest into improving the efficiency and quality of their health care provision and also into prevention and health promotion to achieve better health and better care at lower costs in a sustainable way. Thus, payers need to offer ACOs long-term shared savings contracts linked to quality and patient reported outcomes with regionally-risk adjusted pre- vs. post intervention measurement that support investment in the health of the population (achieving an ROI within this time). In addition, performance measurement and management is a core element of ACO models and data sharing from payers with ACOs a critical ACO contract characteristic to allow for that [10].

*Organizational structure*: Close regional working environment and communication enables a culture of trust between providers, payers and ACO beneficiaries. Facilitating the formation of smaller-sized ACOs as integrators (to allow personal trust and accountability) may therefore lead to higher ACO performance. Nevertheless, these regional, smaller ACOs also need to enrich the local provider know-how with general health science and management capabilities to fulfill its full potential.

*Performance management*: Performance measurement in ACOs is, because of the nature of the contracts, in a first step driven by external audiences. Although, high-value ACOs share this reporting obligation, they primarily use measurement for internal process and performance control and continuous improvement. Efficient ACO performance management systems utilize technical (e.g., health information systems with chronic disease registries and reminders) and personnel (physician extenders) support to improve performance [10].

*Care and coordination processes*: Last but not least, care and coordination processes have to be organized in ACOs. A combination of evidence-based and locally adapted care interventions, activation of patients, shared decision making and self-management support, and interventions beyond health care, including prevention, public health and the social arena, seem to be appropriate to achieve better short and long-term outcomes on the Triple Aim [7].

The variation in performance of ACOs emphasizes the importance to identify good practices employed by successful ACOs, and to find ways for their dissemination. The core principles for ACOs provided above, offer a first start for this endeavor. As re-orienting the model of care towards ACOs has the potential to simultaneously improve quality, costs and patient satisfaction, policy makers in Europe should pay close attention to the ACO reforms in the US. However, the country-specific cultural and value context may not be neglected, as the discussion in the NHS shows, where NHS recent guidance proposed to rename its work on promoting ‘Accountable Care Systems’ to ‘Integrated Care Systems’. This change reflects on the one hand the erroneous perception that Accountable Care Systems automatically bring along a risk of privatization (see [11]) and that on the other hand reforms towards improving integrated care are not just a recent import from the USA, but indeed have a long history in the NHS. The principles of successful ACOs in the US, are described above, nevertheless remain part of the NHS reforms, just as these principles underpin health system reforms in other European countries and abroad. Supporting these reforms however requires dedicated policy. The proliferation of ACOs in the US versus the slow growth in other countries underpins the power of policy to get such a movement started. Such policies need a clear value- and population-based contracting policy path to be implemented, in combination with offering payment models with upfront investment to build a financial foundation for the incremental change necessary for value-based approaches to develop.
